# Autistic traits and proneness to shame and guilt: The mediating role of functional connectivity of cortical midline structures

**DOI:** 10.1017/pen.2025.10004

**Published:** 2026-04-07

**Authors:** Isaac N. Ip, Hey Tou Chiu, Fiona N.Y. Ching, Chun-Kit Law, Esther H.L. Tang, Clayton S.F. Ng, Savio W.H. Wong

**Affiliations:** 1 Centre for Child and Family Science, The Education University of Hong Kong, Hong Kong SAR; 2 Department of Educational Psychology, The Chinese University of Hong Kong, Sha Tin, Hong Kong SAR; 3 Laboratory for Brain and Education, The Chinese University of Hong Kong, Hong Kong SAR; 4 Department of Rehabilitation Sciences, The Hong Kong Polytechnic University, Hong Kong SAR

**Keywords:** autistic traits, functional connectivity, guilt-proneness, resting-state, self-conscious emotions, shame-proneness

## Abstract

Shame and guilt are similar yet distinct self-conscious emotions that often facilitate the attainment of social goals and motivate behaviors that promote social acceptance. Recent studies have shown that individuals with autism or high autistic traits may tend to exhibit higher shame-proneness and lower guilt-proneness. This study examined whether this profile of self-conscious emotions can be explained by the functional organization of the brain using resting-state fMRI. Autistic traits, shame- and guilt-proneness and whole-brain resting-state fMRI data were measured in 45 neurotypical individuals. Our results revealed that the positive association between autistic traits and shame-proneness was mediated by resting-state functional connectivity between the right frontal pole and several regions among the cortical midline structures, including the precuneus, anterior cingulate and posterior cingulate. Additionally, functional connectivity between the right frontal pole and precuneus was found to mediate the negative association between autistic traits and guilt-proneness. These findings highlight the role of the cortical midline structures as a key neural substrate underlying differential experiences of negative self-conscious emotions among individuals with high autistic traits.

Autism spectrum disorder (ASD) is characterized by impaired social-communication interaction and restricted and repetitive behaviors (American Psychiatric Association, 2013). The ASD umbrella term emphasizes the dimensional nature of autism, in which autism can be treated as a set of continuous traits that extend into the general population (Sucksmith et al. 2011; Constantino & Todd, 2003). People with high autistic traits, for instance, would experience similar difficulties in social communication although they are not clinically diagnosed with the disorder (Constantino & Todd, 2003).

Autistic individuals are also known for difficulties in social cognition, such as Theory of Mind (ToM) and perspective taking (Baron-Cohen et al., 1985; Frith, 2001), which make it difficult for one to attribute thoughts and feelings to one-self and to others (Mazza et al., 2017). As such, individuals with autism or high autistic traits may more likely violate social norms and commit transgressions because they appraise social situations differently. This has implications on their experience of negative self-conscious emotions (SCEs), which refer to a class of emotions elicited by the breaking of social norms or rules, such as shame and guilt (Tangney & Dearing, 2004).

The study of negative SCEs in autistic individuals or high autistic traits has clinical importance as atypical processing of SCEs is associated with maladaptive behaviors and depression (De Rubeis & Hollenstein, 2009; Tangney & Dearing, 2004). Given high rates of co-occurring affective disorders among autistic individuals (Hudson et al., 2019; Lugo-Marin et al., 2019), it is important to understand how individuals with high autistic traits experience negative SCEs, and the mechanisms that contribute to potential disturbances in the processing of negative SCEs (Davidson et al., 2017).

## Negative self-conscious emotions: shame and guilt

Shame and guilt are moral emotions that serve to motivate us to act in accordance with social norms and personal standards (Else-Quest et al., 2012). These cognitively complex emotions differ from basic emotions (e.g., anger, fear, joy, sadness; Izard, 2007) by a number of characteristics (Tracy & Robins, 2004). First, SCEs require self-awareness and self-representations, meaning that they arise only when individuals become aware that they have (failed to) live up to some actual or ideal self-representation. Second, SCEs require causal attribution and occur only when an eliciting event is attributed internally to the self. Finally, whereas basic emotions promote biological goals, SCEs promote the attainment of social goals and motivate behaviors that promote social acceptance (Tracy & Robins, 2004).

Nevertheless, these SCEs do differ from each other. Shame is characterized by a sense of shrinking and worthlessness and involves an affective reaction to a devaluation of the self. (Muris et al., 2014; Tangney & Tracy, 2012). It is generated by attributing the failure or transgression to the global self and is coupled with motivation tendency to avoid or withdraw (Muris et al., 2014; Tangney & Tracy, 2012). Guilt is characterized by a sense of tension and remorse and involves a preoccupation with the transgression or failure instead of the self. It is generated by attribution to one’s specific behavior and is associated with motivation tendency to repair (Muris et al., 2014; Tangney & Tracy, 2012). Experiences in shame and guilt are adaptive for attaining social goals, however, these emotions can be dysregulated in that they either 1) dominate the way one responds emotionally or 2) when they are absent and fail to promote their adaptive functions (Muris et al., 2014). Taken together, shame and guilt have important roles in maintaining social relationships and the navigation of social interactions. On the one hand, guilt after a transgression can help maintain social relationships, but on the other hand, too much of shame or a lack of guilt could inhibit prosocial behaviors and formation of close social relationships (Tangney & Dearing, 2004).

SCEs typically develop later in life than basic emotions, which coincide with an individuals’ acquisition of advanced social cognition abilities (Muris et al., 2014). Importantly, these social cognitive abilities such as the capacity for self-awareness, stable self-representations, and an awareness of social rules, are critical to the experience of SCEs (Lewis, 2007; Tangney & Tracy, 2012). Theory of Mind (ToM), which is the ability to understand others’ mental states and predict others’ behaviors (Saxe et al., 2004), is key to the self-evaluative process that elicits SCEs, as one has to perform evaluations based on others’ view of them (Leary et al., 2007). For example, an individual may feel guilt or shame because they have beliefs about how other people view their behavior. Indeed, studies have shown associations between ToM abilities and levels of understanding of shame and guilt (Misailidi, 2018, 2020). Interestingly, levels of ToM may contribute towards different proneness to shame and guilt (Yang et al., 2010). In an experimental study, undergraduate participants were randomly assigned to shame, guilt and control affect-induction conditions and were then engaged in a perspective-taking task. Compared with participants in the control condition, participants experiencing guilt performed better in the perspective-taking task, but participants experiencing shame performed worse perspective-taking (Yang et al., 2010). The results provided initial evidence that ToM abilities could be differentially associated with shame and guilt.

## Shame- and guilt-proneness in autism

Autistic individuals are known for their difficulties in social cognition (Baron-Cohen, 1990; Frith, 2001; Chung et al., 2014). This has been demonstrated through lower performances among autistic versus NT individuals on behavioral assessments (e.g., Baron-Cohen et al., 2001; Dziobek et al., 2006; Dziobek et al., 2008) and naturalistic based assessments (e.g., Ponnet et al., 2008; Heavey et al., 2000) of ToM. This is also reflected by positive associations between autism severity and ToM difficulties (Happe & Frith, 2014). However, it should be noted that ToM difficulties may not be universal among autistic individuals (Gernsbacher & Yergeau, 2019) with considerable variability in performance, as shown in autistic adults (Brewer et al., 2017). Nevertheless, studies have also assessed autistic traits and ToM in the general population using trait-based measures, for example, Autism Quotient (AQ; Baron-Cohen et al., 2001, Hoekstra et al., 2011), Broad Autism Phenotype Questionnaire (BAPQ; Hurley et al., 2007). For example, Gökçen et al. (2016) found that adults and adolescents with higher AQ scores had greater difficulty in identifying mental states on a video-based mentalizing task. Similarly, higher BAPQ scores was associated with poorer performance on ToM tasks in younger (aged 18–46) and older (aged 60–91) adults (Stewart et al., 2020). In sum, such studies indicate that in the absence of a clinical diagnosis, individuals with higher autistic traits could be more prone to difficulties in ToM and/or perspective taking.

The potential difficulties in social cognition in autistic individuals suggest that they may also experience SCEs differently. Early studies reported impaired recognition of shame (Heerey et al., 2003) and a weaker ability to produce coherent and contextually appropriate descriptions of shame and guilt among autistic children (Losh & Capps, 2006). More recent studies using behavioral or scenario-based measures suggest that individuals with autism or high in autistic traits showed a different tendency to experience shame and guilt than NT individuals. For instance, Davidson et al. (2017) measured autistic traits and proneness to shame and guilt using the Social Responsiveness Scale (SRS-2; Constantino & Gruber, 2012) and Test of Self-Conscious Affect (TOSCA-3; Tangey et al. 2000), respectively, in a group of NT adults. They found that those who scored higher in autism traits (overall score ≥ 60) had higher shame-proneness but lower guilt-proneness than those who scored lower in autism traits. A similar study that measured proneness to shame and guilt using the children version of TOSCA found that autistic children had lower guilt-proneness, but comparable shame-proneness than NT children (Davidson et al., 2018). Meanwhile, van Trigt et al. (2023) induced self-conscious emotions in children using a broken toy paradigm, in which the experimenter’s toy was “broken” by the children, and observed their reactions. They found that children as young as 2 to 5 years old displayed more shame-like avoidance if their autistic traits were higher.

Certain aspects of shame behavior may be comparable among autistic and NT individuals. Using the Guilt and Shame Proneness Scale (GASP; Cohen & Wolf, 2011), Gaziel-Guttman et al. (2022) found that autistic adults showed lower levels of shame, in terms of lower negative self-evaluation but similar withdrawal tendency in comparison to NT individuals. This suggests a discrepancy between internal and external shame behavior. In other words, autistic individuals show lower emotional self-impact upon experiencing shame, but their external behavior, that is, withdrawal, remains socially adaptive. This recent study suggests that differences in expressions of shame in autism is complex and not uniform. Although inconsistent findings exist, the majority of studies so far, suggest that individuals with high autistic traits tend to experience higher levels of shame. As for guilt-proneness, however, individuals with high autistic traits tend to experience lower levels of guilt. Further research is still required to understand the mechanisms affecting the experiences of SCEs in autism.

One particular mechanism that has yet to be explored is the neural substrates of shame and guilt-proneness in individuals with autism or high autistic traits. This is an area worthy of investigation as neuroimaging studies may help tease apart the various processes that underlie the processing of SCEs (Jankowski & Takahashi, 2014) and inform us about the atypical cognitive processes that contribute to dysregulations of SCEs in autism.

## Role of the cortical midline structures (CMS) in shame- and guilt-proneness and autism

The cortical midline structures (CMS), including the medial prefrontal cortex (mPFC), the anterior cingulate cortex (ACC), the posterior cingulate cortex (PCC), and the precuneus (PC), may be key substrates that underlie the altered profile of high shame-proneness and low guilt-proneness in individuals with high autistic traits. The CMS regions are implicated in several key functions that support social interactions and relationships, including self-referential processing and social cognition (Northoff et al., 2006; Northoff & Bermpohl, 2004; Sebastian et al., 2008). Neuroimaging studies of ToM tasks have shown the involvement of fronto-temporo-parietal networks, consisting of CMS like the mPFC, PCC, PC, and additionally the temporo-parietal junction (TPJ), temporal poles (TP), and superior temporal sulci (STS) (Schurz et al., 2014; Gallagher & Frith, 2003). The CMS also has dense connections with subcortical structures, for example, the insula and the brain stem, which are regions important for interoception (Northoff et al., 2006; Nagai et al., 2004; Craig, 2004).

Specifically, fMRI studies on shame and guilt in NT individuals have shown activations that overlap with the CMS (e.g., ACC, PCC) (Michl et al., 2014, Moll et al., 2007), ToM network (e.g., dorsal mPFC, STS, TPJ) (Wagner et al., 2011; Takahashi et al., 2004), and emotional processing (e.g., insula, amygdala) (Wagner et al., 2011; Yu et al., 2014). Given the theoretical distinctions between shame and guilt, studies have contrasted the two negative SCEs using scenario-based emotion-induction paradigms. One study highlights the role of the mPFC in guilt compared to shame, whereas the mid-temporal gyrus (MTG) was activated in shame compared to guilt (Takahashi et al., 2004). Others, however, have found greater activations in the mPFC, ACC and PCC for shame compared to guilt (Michl et al., 2014). More recent studies have used paradigms inducing shame and guilt in interpersonal contexts. Zhu et al. (2019) used multivariate pattern analysis (MVPA) and demonstrated differential neural representations of guilt versus shame which included the CMS, for example, dmPFC, ventral ACC, and regions related to cognitive control (i.e., ventrolateral PFC, dorsolateral PFC). Structural MRI studies have complemented these findings and have shown that anatomical differences in the CMS, for example, larger ACC, was associated with compensatory behavior in a guilt-inducing task (Yu et al., 2014), and that higher levels of proneness to shame were associated with smaller amygdala and thinner PCC (Whittle et al., 2016). In sum, findings so far have not been consistent to distinguish neural substrates that are unique to shame and guilt, likely due to the subtle conceptual difference between the two emotions. However, they have continued to highlight the importance of the CMS in self-processing and ToM, which both play roles in negative SCEs like shame- and guilt-proneness.

The CMS are highly represented by the default mode network (DMN), a core brain system frequently implicated in autistic individuals (e.g., Patriquin et al., 2016; Padmanabhan et al., 2017). The DMN is comprised of a set of interconnected brain regions, including primarily the mPFC, PCC, and bilateral inferior parietal lobules (IPL), that are active when an individual is not engaged in any specific task or focused on the outside world (Whitfield-Gabrieli & Ford, 2012). The DMN is suggested to play a central role in the normal functioning of various cognitive processes, including autobiographical memory, self-awareness, and social cognition (Raichle, 2015). Task-based and resting-state fMRI studies have reported reduced activity or connectivity in the DMN in autistic individuals (Pantelis et al., 2015; Assaf et al., 2010; Jung et al., 2014; von Dem Hagen et al., 2013), which is associated with lower socio-emotional cognition abilities (Doyle-Thomas et al., 2015; Kana et al., 2015; Assaf et al., 2010). Furthermore, negative correlations have been consistently reported between autistic traits and functional connectivity of the DMN regions, for example, mPFC (Jung et al., 2014; von Dem Hagen et al., 2013; Monk et al., 2009), ACC (Assaf et al., 2010), PCC (Yerys et al., 2015) in either autistic and/or NT individuals. These studies point to altered functional connectivity in the DMN, including the CMS, as prominent neurobiological features that underlie the individual differences of social difficulties in autistic and NT individuals.

## The present study

Given the above, the marked overlap between brain regions associated with atypical connectivity in autism and those of social and self-referential cognitive processes, that is, CMS, which closely support the processing of negative SCEs, that is, shame and guilt, pose us with the question of whether the functional organization of the brain can explain the link between autistic traits and SCEs. To the best of our knowledge, no study has explored whether resting-state functional connectivity (rs-FC) can be a physiological mediator of the association between autistic traits and individual differences in shame- and guilt-proneness.

Taking the continuum view of autism, autistic traits can be associated with socio-cognitive difficulties regardless of whether an autism diagnosis exists or not (Constantino & Todd, 2003). Davidson et al. (2017) first demonstrated the differential association of shame and guilt-proneness to autistic traits, using the SRS-2. However, whether this association is replicable across other well-established autistic trait measures, such as the AQ and BAPQ, is unclear. Ingersoll et al. (2011) reported moderate correlations between the SRS, AQ, and BAPQ in NT adults. Although a consensus on the optimal method for measuring autistic traits has yet to emerge (Ingersol & Wainer, 2014), both AQ and BAPQ have been widely used and assess a similar underlying construct (Ingersoll et al., 2011) with distinct focuses. While the AQ primarily measures difficulties in social functioning, including communication and attention switching (Hoekstra et al., 2011), the BAPQ evaluates a triad of traits specific to the boarder autism phenotype: rigid behaviors, aloofness and pragmatic language difficulties (Hurley et al., 2007). Accordingly, the first goal of the study was to examine the associations of autistic traits in NT adults with proneness to shame and guilt, through the administration of TOSCA-3 with measures of both AQ and BAPQ. We expected to extend and replicate findings from previous studies that people with higher autistic traits will show higher shame-proneness and lower guilt-proneness.

The second goal of the study was to explore whether the rs-FC of the CMS would mediate the relations between autistic traits and shame- and guilt-proneness. Since no previous neuroimaging studies have investigated this before, nor did we have well-defined region of interests (ROIs) associated with shame- and guilt-proneness, we adopted a hypothesis-free data-driven approach (i.e., Intrinsic Connectivity Contrast, ICC) to identify common regions of functional connectivity that can be predicted by shame, guilt and autistic traits. The ICC approach has advantages in its sensitivity to detect voxel-wise whole-brain connectivity patterns, without the need for a-priori ROIs (Martuzzi et al., 2011; Scheinost et al., 2012). As the ICC is largely an exploratory approach, follow-up seed-based connectivity (SBC) analyses with the clusters derived by the ICC analysis were used to determine the specific regions that contributed to the differences in global functional connectivity. This two-step approach is increasingly being used to characterize the profile of brain-based neural markers of various psychopathologies, for example, anxiety (Xu et al., 2021), borderline personality disorder (Sarkheil et al., 2020). If functional connectivity of any CMS is a physiological mechanism of autistic traits and shame- or guilt-proneness, they should at least be identified by this method. Finally, we tested the mediating effect of these specific functional connectivity on the relationship between autistic traits and proneness to shame and guilt.

## Methods

### Participants

As no prior study has examined the association between autistic traits with shame- and guilt-proneness as a mediation model, power analysis was conducted using G*power 3.1 software (Faul et al., 2009) based on the effect size obtained from Gaziel-Guttman et al. (2022), where levels of shame-proneness among NT and autistic young adults were examined. Based on Cohen’s *d* = 0.88, with an alpha = 0.05 and power = 0.8 for linear multiple regression analyses, the sample size required was 43.

Forty-five Chinese young adults (20 females, age: M = 22.0, SD = 3.88) completed a resting state fMRI scan and a set of questionnaires measuring their autistic traits and proneness to shame and guilt as part of a larger study. Participants were recruited by means of convenient sampling through advertisements in university mass mail, social media platforms, and word of mouth. Inclusion criteria were right-handedness, age between 18 and 35, normal or corrected to normal vision, MRI-compatibility, capability of giving informed consent and native in Chinese language. Exclusion criteria were presence or history of neurological or psychiatric disorder, special educational needs, and pregnancy. Written informed consent was obtained from all participants.

### Questionnaire measures

#### Shame- and guilt-proneness

Proneness to shame and guilt were measured by the Test of Self-Conscious Affect 3 (TOSCA-3; Tangney et al., 2000). The TOSCA-3 is a scenario-based measure of self-conscious emotions consisting of 16 scenarios and response sets (e.g., “You are driving down the road and you hit a small animal: 1. You would think: ‘I’m terrible.’ (Shame); 2. You’d feel bad you hadn’t been more alert driving down the road.” (Guilt). Responses are rated on a scale from 1 (“not likely”) to 5 (“very likely”). A Chinese version of TOSCA-3 was used (Gao et al., 2013). Subscales of shame- and guilt-proneness both demonstrated good reliability (αs: shame = .79, guilt = .76).

#### Autistic traits

Two measures of autistic traits were used. The short version of Autism Spectrum Quotient (AQ-S; Hoekstra et al., 2011) was used to assess participants’ autistic traits (Baron-Cohen et al. 2001). While there are 50 items in the original version, the AQ-S is a 28-item self-report questionnaire consisting of five factors, namely “social skills,” “routine,” “switching,” “imagination,” and “numbers/patterns.” Participants respond to each statement on a 4-point Likert scale (1 = definitely agree; 2 = slightly agree; 3 = slightly disagree; 4 = definitely disagree). Item scores are summed, with a minimum score of 28 and a maximum of 112. A Chinese version of AQ-S was adopted and demonstrated good reliability (α = .78). While the AQ-S is not a diagnostic instrument (Hoekstra et al., 2011), 18 out of 45 participants had scores that exceeded the suggested stringent cut-off of 70.

The Broad Autism Phenotype Questionnaire (BAPQ; Hurley et al., 2007) was used to measure personality and language characteristics, which are subclinical and qualitatively similar to the defining features of autism. The 36-item BAPQ consists of three dimensions: aloof personality, defined as “a lack of interest in or enjoyment of social interaction,” rigid personality, defined as “little interest in change or difficulty adjusting to change,” and pragmatic language problems, defined as “deficits in the social aspect of language that result in difficulties communicating effectively or in holding a conversation.” Participants respond to each statement on a 6-point rating scale (1 = very rarely, 2 = rarely, 3 = occasionally, 4 = somewhat often, 5 = often, 6 = very often). A total score was calculated by summing all 36 items, with a minimum score of 36 and a maximum of 216. A translated Chinese version of the BAPQ was adopted for this study. The BAPQ demonstrated excellent reliability (*α* = .90).

### fMRI data acquisition

Scanning was performed using a Siemens MAGNETOM Prisma 3T MR scanner. Five minutes of resting state BOLD function scans were acquired for each subject with an echoplanar pulse sequence [TR/TE = 1240/30 ms; FA = 63°; FOV = 208 × 208 mm; matrix size = 84 × 84; 54 axial slices; voxel size = 2.5 mm isotropic; slice order = interleaved]. A total of 242 volumes were acquired with a through-plane acceleration factor (SMS) of 3. Foam padding was used as far as possible to comfortably restrict head movement. An anatomical reference image was acquired for each subject using a magnetization-prepared rapid acquisition gradient echo sequence (MPRAGE) [TR/TE = 1900/2.15 ms; FA = 9°; FOV = 256 × 256 mm; 176 axial slices; voxel size = 1 mm isotropic; slice order = ascending; acceleration factor (GRAPPA) = 2]. Participants laid in supine position inside the MRI scanner. They were shown a fixation cross and were instructed to keep their head still, stay focused on the fixation cross, and not to think about anything in particular. The procedure lasted for five minutes.

### fMRI data preprocessing and denoising

Preprocessing was performed using the CONN toolbox version 20.b (Whitfield-Gabrieli & Nieto-Castanon, 2012) using the default preprocessing pipeline for volume-based analyses (indirect normalization to MNI-space). The following steps were performed: functional realignment and unwarp, outlier identification, indirect segmentation and normalization, and smoothing. Field maps were used for susceptibility distortion correction during the realignment and unwarp step. The CONN toolbox identifies potential outlier scans using the observed BOLD signal and subject-motion in the scanner. Specifically, potential outliers were identified when global BOLD signal changes were above five standard deviations or when framewise displacement was above 0.9 mm. During indirect segmentation and normalization, the anatomical image was first co-registered to the functional images, then segmented into gray matter, white matter, and cerebrospinal fluid (CSF) tissue classes based on the ICBM tissue probability maps and normalized onto standard MNI space (Whitfield-Gabrieli & Nieto-Castanon, 2012). The non-linear transformation resulting from this normalization procedure was applied to the functional images, which were then smoothed with a 4 mm FWHM Gaussian filter. The functional data were denoised using CONN’s default denoising pipeline. This involved linear regression of potential confounding effects in the BOLD signal, including noise components from white matter and CSF, estimated subject-motion parameters (6 motion parameters plus first-order derivatives), and scrubbing of noise components during outlier identification, followed by temporal band-pass filtering (0.008 Hz and 0.09 Hz). After inspection of quality assurance parameters (e.g., the maximum and mean extent of motion, number of invalid scans), none of our participants were excluded from the analyses.

### Statistical analyses

#### Questionnaire analyses

To test whether shame- and guilt-proneness would differentially associate with the AQ and BAPQ, zero-order and part correlations were performed. As shame- and guilt-proneness often highly correlate with each other, some recommend its associations with other variables analyzed as partial correlations of guilt-free shame and shame-free guilt (e.g., correlation with shame while controlling for guilt, vice versa) (Rüsch et al., 2007). Thus, we adopt the same approach for the analyses of the behavioral data and subsequent functional connectivity analyses. Inspections of the questionnaire measures using Shapiro-Wilks tests indicated that none of the scores violated normality assumptions: *W*(45) = .976, *p* = .481 for AQ; *W*(45) = .954, *p* = .071 for BAPQ, *W*(45) = .964, *p* = .176 for proneness to shame, and *W*(45) = .958, *p* = .101 for proneness to guilt. All statistical analyses were evaluated with the level of significance set at *p* <. 05.

#### Functional connectivity analyses

To identify ROIs, ICC maps were computed to assess hypothesis-free whole-brain connectivity for each voxel in the brain. ICC is computed as the root mean square of correlation coefficients between each individual voxel and all other voxels in the brain. The 1st-level analysis yields a map for each participant where a higher value represents the strength of connectivity of that voxel with the rest of the brain and thus a network measure of node centrality (i.e., from graph theory). Two separate 2nd-level GLMs that include proneness to shame and guilt, with either BAPQ or AQ as covariates were used to delineate ROIs where their node centrality would be associated with individual differences in autistic traits and proneness to shame and guilt.

Next, the obtained ROIs were used for follow-up SBC analyses to probe further into their functional connectivity with specific brain regions. The 1st-level analysis produces SBC map for each participant, indicating how well the time series of each voxel in the brain correlates with the time series of the seed region. Again, two separate GLMs with the same 2nd-level covariates as the ICC analysis were performed, in order to identify functional connectivity that would be associated with individual differences in autistic traits and proneness to shame and guilt. For all 2nd-level analyses, cluster-level correction for multiple comparisons were based on Gaussian random field theory (*p* < .05, FWE-corrected). Both ICC and follow-up SBC analyses were conducted using the CONN toolbox.

#### Mediation analyses

**Figure 1.**
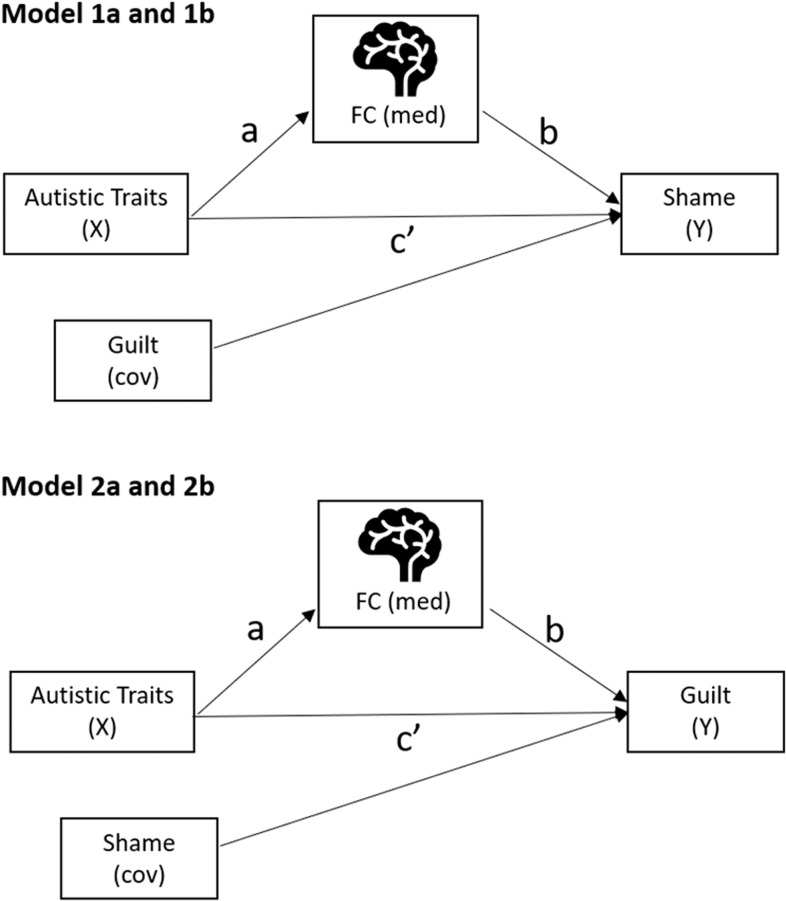
Conceptual diagram of the mediation models. *Note:* A total of four models were tested. These models tested whether the identified functional connectivity (FC) from the seed-based connectivity analyses, mediated the effect of autistic traits on shame- or guilt-proneness. Model 1a: X = AQ, Y = shame, cov = guilt. Model 1b: X = BAPQ, Y = shame, cov = guilt. Model 2a: X = AQ, Y = guilt, cov = shame. Model 2b: X = BAPQ, Y = guilt, cov = shame. In all models, med = functional connectivity. Mediation effect (or indirect effect) is represented by the product of path coefficients *a* and *b*.

To test whether functional connectivity would explain the association between autistic traits and proneness to shame and guilt, the following mediation models were performed. The independent variable (X) was AQ or BAPQ and the mediator (med) was functional connectivity (FC) identified through SBC analyses. When the dependent variable (Y) was proneness to shame, the covariate (cov) was guilt; whereas if the dependent variable (Y) was proneness to guilt, the covariate (cov) was shame (see Fig. 1). Thus, a total of four models were performed. The path coefficient *a* reflects the effects of X on the mediator, while *b* reflects the effects of the mediator on Y. The path coefficient *c’* reflects the direct effect of X on Y after controlling for the mediator. The total effect is the effect of X on Y without controlling for the mediator. The test of mediation was performed using PROCESS version 4.3, which involves estimating the indirect effect (i.e., the product of path coefficients *a* and *b*). The significance of the indirect effect was tested using bootstrap confidence intervals with 5000 random samples. Statistical significance is indicated when the confidence intervals do not contain zero (Hayes, 2018). The effect size of mediation, that is, the proportion mediated (P_M_), is calculated as the ratio of the indirect effect to the total effect.

## Results

### Autistic traits and proneness to shame and guilt

Means and standard deviations of questionnaire measures are shown in Table 1. Zero-order and part correlations between AQ, BAPQ, shame-proneness, and guilt-proneness are shown in Tables 2 and 3, respectively (for partial correlation plots, see *Supplementary Material*). As expected, a positive part correlation between shame-proneness (controlling for guilt-proneness) and AQ score was observed, *r*(43) = .382, *p* = .005. Similarly, there was a positive part correlation between shame-proneness (controlling for guilt-proneness) and BAPQ score, *r* (43) = .469, *p* = .001. Thus, higher scores in AQ and BAPQ were associated with higher levels of shame-proneness.

**Table 1.** Means and standard deviations of questionnaire measures

Measures	Range	*M (SD)*
1. Shame-proneness	16–80	50.2 *(9.2)*
2. Guilt-proneness	16–80	61.7 *(7.3)*
3. AQ	28–112	70.1 *(8.8)*
4. BAPQ	36–216	119.3 *(19.8)*

*Note:* Ratings for proneness to shame and guilt ranged from 1 to 5, with higher scores indicating greater proneness to the emotion. For autistic trait measures, higher scores indicated greater autistic traits. Range is the minimum and maximum score for the particular scale. AQ, Autism Quotient (Short); BAPQ, Broad Autism Phenotype Questionnaire.

**Table 2.** Zero-order (top triangle) and part correlations (bottom triangle) between AQ, BAPQ, and shame-proneness

	1	2	3
1. Shame-proneness	–	.341*	.339*
2. AQ	.383*	–	.641***
3. BAPQ	.469**	.648***	–

*Note*: AQ, Autism Quotient (Short); BAPQ, Broad Autism Phenotype Questionnaire. Part correlation controlling for guilt-proneness.

*p < .05, **p < .01, ***p < .001, one-tailed.

**Table 3.** Zero-order (top triangle) and part correlations (bottom triangle) between AQ, BAPQ, and guilt-proneness

	1	2	3
1. Guilt-Proneness	–	.038	−.096
2. AQ	−.188	–	. 641
3. BAPQ	−.356*	.594***	–

*Note:* AQ, Autism Quotient (Short); BAPQ, Broad Autism Phenotype Questionnaire. Part correlation controlling for shame-proneness.

*p < .05, one-tailed.

A negative part correlation between guilt-proneness (controlling for shame-proneness) and BAPQ score was observed, *r* (43) = −.356, *p* = .018, so that higher scores in BAPQ were associated with lower levels of guilt-proneness. However, the part correlation between guilt-proneness and AQ total score was not significant.

### Functional connectivity analyses

#### Intrinsic connectivity contrast

**Figure 2.**
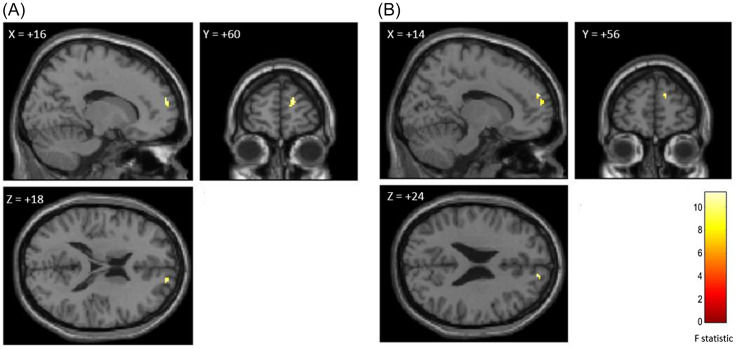
ICC results showing overlapping region of right frontal pole predicted by shame- and guilt-proneness and autistic traits. *Note:* a) ICC of ROI when autistic traits were measured by AQ. b) ICC of ROI when autistic traits were measured by BAPQ. Statistical threshold for both sets of results were set at *p* < .05 with FWE-correction. Images are thresholded at *p* < .001 with *k* = 10 voxels for presentation purpose.

Two separate 2nd-level GLM identified overlapping regions within the right frontal pole (rFP) where ICC was predicted by autistic traits and shame- and guilt-proneness. The first GLM, which included AQ and shame- and guilt-proneness as covariates, identified a region within the rFP (cluster size: 17 voxels, MNI peak: [+16, +60, +18] (BA 10), *p*-FWE = .046; see Fig. 2a). Similarly, the second GLM, which included BAPQ and shame- and guilt-proneness as covariates, identified a region within the rFP (cluster size: 22 voxels, MNI peak: [+14, +56, +24] (BA 9), *p*-FWE = .009; see Fig. 2b).

#### Seed-based connectivity analysis

**Figure 3.**
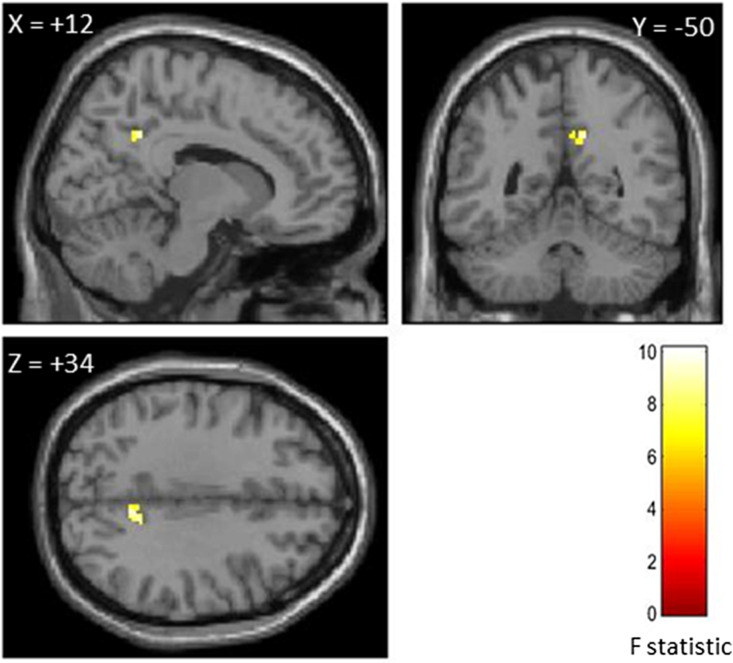
Region in which functional connectivity with the right frontal pole was predicted by shame-and guilt-proneness and autistic traits measured by AQ. *Note:* Seed-based connectivity (SBC) analyses showing correlation between the precuneus (PC) with rFP ROI identified from ICC analysis [+12, −50, +34] (see Fig 2a). Statistical threshold for SBC results was set at *p* < .05 with FWE-correction. Images are thresholded at *p* < .001 uncorrected with *k* = 20 voxels for presentation purposes.

**Figure 4.**
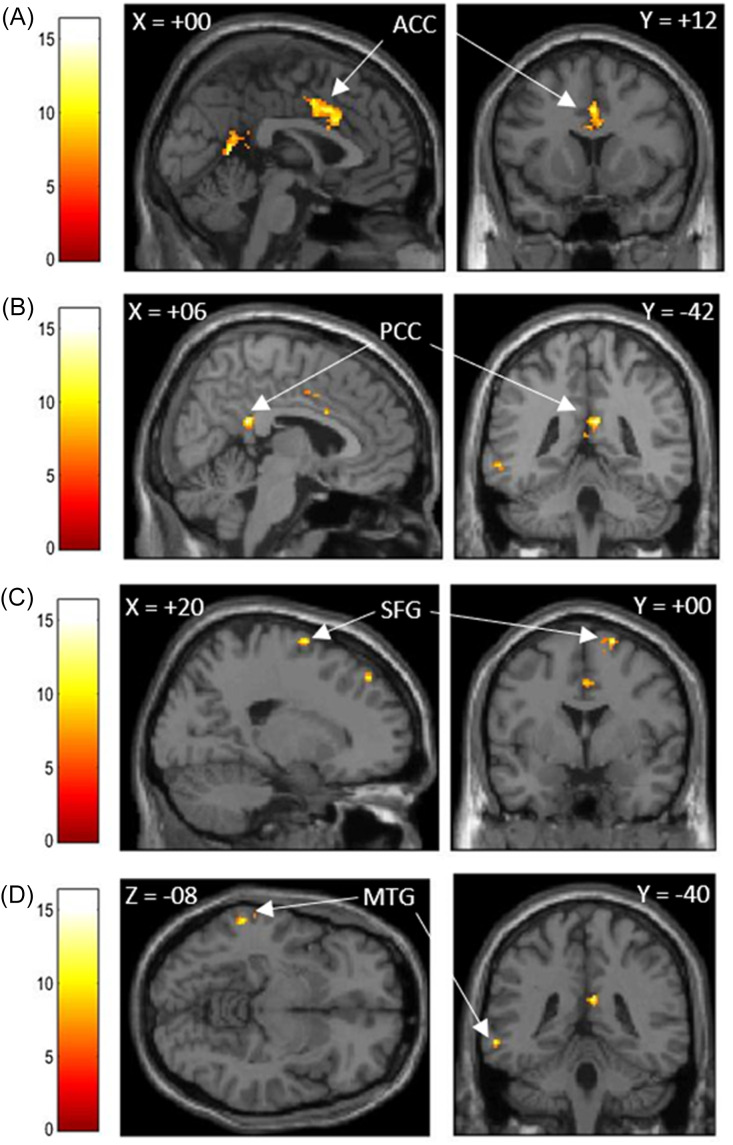
Regions in which functional connectivity with the right frontal pole was predicted by shame-and guilt-proneness and autistic traits measured by BAPQ. *Note:* Seed-based connectivity (SBC) analyses showing areas correlated with rFP ROI identified from ICC analysis [+14, +56, +24] (see Fig 2b): A) anterior cingulate cortex (ACC), B) posterior cingulate cortex (PCC), C) right superior frontal gyrus (SFG), D) left middle frontal gyrus (MFG). Statistical threshold for SBC results was set at *p* < .05 with FWE-correction. Color bar denotes F-statistic range. Images are thresholded at *p* < .001 uncorrected with *k* = 20 voxels for presentation purposes.

Using the corresponding ROI (i.e., rFP) identified by the ICC analyses as seeds, two separate 2nd-level GLM that included autistic traits, shame-, and guilt-proneness as covariates were performed. Firstly, AQ scores were found to associate with the functional connectivity with a region within the precuneus (PC) (Fig. 3, Table 4). Secondly, BAPQ scores were found to associate with the functional connectivity with regions in the ACC, PCC, right superior frontal gyrus (rSFG), and left posterior mid temporal gyrus (lPMTG) (Fig. 4, Table 4).

**Table 4.** List of brain regions from SBC analyses that correlated with seeds identified from ICC analyses

	Brain regions	BA	Cluster size (voxels)	Peak voxel *F*-value	MNI coordinates		
					X	y	z
a) seed: rFP [+16, +60, +18]	Precuneous	31	48	9.31	+12	−50	+34
b) seed: rFP [+14, +56, +24]	Anterior cingulate cortex	32	255	22.20	+00	+12	+38
	Posterior cingulate cortex	23	118	15.31	+6	−42	+22
	Superior frontal gyrus right	6	50	17.85	+20	+0	+70
	Middle temporal gyrus left	21	41	13.42	−62	−40	−8

*Note:* a) right frontal pole seed identified from ICC analysis with AQ, shame- and guilt-proneness as predictors. b) right frontal pole seed identified from ICC analysis with BAPQ, shame- and guilt-proneness as predictors. *p* < .05, FWE-corrected.

### Mediation analyses

Having identified regions of functional connectivity in the brain that were associated with autistic traits, shame- and guilt-proneness (i.e., AQ: PC; BAPQ: ACC, PCC, rSFG, lPMTG), we further conducted mediation analysis to test whether functional connectivity could be a mechanism explaining the differential association of shame- and guilt-proneness for people with high autistic traits.

With respect to shame-proneness, functional connectivity between the rFP and PC significantly mediated the positive association between shame-proneness and autistic traits measured by AQ (Table 5, Model 1a). Further, functional connectivity between the rFP and ACC, PCC, and rSFG significantly mediated the positive association between shame-proneness and autistic traits measured by BAPQ (Table 5, Model 1b).

**Table 5.** Path coefficients of mediation models predicting shame-proneness

		Total effect	Direct effect	Indirect effect	95% CI		Mediation effect size
		*c*	*c’*	*a* x *b*	LL	UL	P_M_
*Model 1a*							
X	AQ	0.321*	0.17				
Mediator	fc_PC			0.151	**0.025**	**0.314**	0.470
*Model 1b*					* *	* *	
X	BAPQ	0.395**	0.195				
Mediators	fc_ACC			−0.289	−**0.476**	−**0.151**	−0.732
	fc_PCC			0.255	**0.071**	**0.527**	0.646
	fc_rSFG			0.132	**0.001**	**0.328**	0.334
	fc_lPMTG			0.103	−0.053	0.325	0.261

*Note:* AQ, Autism Quotient (Short); BAPQ, Broad Autism Phenotype Questionnaire; fc, functional connectivity; PC, precuneus; ACC, anterior cingulate cortex; PCC, posterior cingulate cortex; rSFG, right superior frontal gyrus; lPMTG, left posterior mid temporal gyrus. Bolded confidence interval excludes zero and indicates statistical significance.

*p < .05, **p < .01, two-tailed.

Similarly, functional connectivity between the rFP and PC mediated the negative association between guilt-proneness and autistic traits measured by AQ (Table 6, Model 2a). However, out of the four clusters identified from the ICC analysis, only functional connectivity between the rFP and rSFG significantly mediated the negative association between guilt-proneness and BAPQ (Table 6, Model 2b).

**Table 6.** Path coefficients of mediation models predicting guilt-proneness

		Total effect	Direct effect	Indirect effect	95% CI		Mediation effect size
		*c*	*c’*	*a* x *b*	LL	UL	P_M_
*Model 2a*							
X	AQ	−0.168	0.022				
Mediator	fc_PC			−0.19	−**0.352**	−**0.034**	1.13
*Model 2b*							
X	BAPQ	−0.318*	0.185				
Mediators	fc_ACC			0.066	−0.21	0.362	−0.208
	fc_PCC			−0.092	−0.35	0.01	0.289
	fc_rSFG			−0.307	−**0.541**	−**0.102**	0.965
	fc_lPMTG			−0.17	−0.424	0.061	0.535

*Note:* AQ, Autism Quotient (Short); BAPQ, Broad Autism Phenotype Questionnaire; fc, functional connectivity; PC, precuneus; ACC, anterior cingulate cortex; PCC, posterior cingulate cortex; rSFG, right superior frontal gyrus; lPMTG, left posterior mid temporal gyrus. Bolded confidence interval excludes zero and indicates statistical significance.

*p < .05, two-tailed.

## Discussion

To the best of our knowledge, this is the first study that investigated whether profiles of high shame-proneness and low guilt-proneness in individuals with high autistic traits could be explained by the functional organization of the brain. We first replicated previous findings by showing that participants with higher autistic traits had higher levels of shame-proneness. Higher autistic traits measured by the BAPQ were also associated with lower levels of guilt-proneness. Importantly, a hypothesis-free data-driven ICC analysis of rs-FC data revealed differential connectivity of the right frontal pole, which was associated with autistic traits, shame- and guilt-proneness. This guided us in our follow-up seed-based analyses which identified several regions among the CMS (i.e., PC, ACC, PCC, rSFG), that mediated the positive and negative associations between autistic traits and shame- and guilt-proneness, respectively. Together, these findings provide initial evidence of the role of CMS functional connectivity, regions closely relevant to social cognition, as a neurobiological mechanism underpinning the differential experience of negative SCEs among individuals with varying levels of autistic traits.

In two separate ICC analyses, we identified two overlapping regions in the right frontal pole that showed changes in global connectivity when predicted by individual differences in shame- and guilt-proneness and autistic traits. This is in line with past studies that have identified associations of the frontal pole region (BA 9/10) with the processing of guilt (Takahashi et al., 2004; Moll et al., 2007), shame (Zhu et al., 2019), and social versus nonsocial emotions (Gilead et al., 2016). Moreover, the CMS regions subsequently identified through our seed-based analysis are also consistent with past studies on the functional and anatomical correlates of self-conscious emotions (e.g., PC: Gilead et al., 2016; Michl et al., 2014), shame (e.g., ACC: Michl et al., 2014; PCC: Michl et al., 2014, Whittle et al., 2016), and guilt (e.g., ACC, Yu et al., 2014). The results also align with studies showing the involvement of the SFG and the MFG regions in processing guilt (e.g., Zhu et al., 2019; Michl et al., 2014) and shame (e.g., Michl et al., 2014). Crucially, the identified CMS regions are within the DMN, and corroborates with consistent associations between atypical connectivity in the midline core, that is, the mPFC-PCC, and social impairment among individuals with high autistic traits or autism (Yerys et al., 2014; Jung et al., 2014; Assaf et al., 2010). Together, the findings support the literature showing the role of CMS in self-referential processing, which may underlie both shame and guilt-proneness, and the social difficulties in autistic individuals.

Consistent with the findings of Davidson et al. (2017), our results indicate that higher autistic traits, measured by both the AQ and BAPQ, are associated with greater shame-proneness. Furthermore, higher traits on the BAPQ were linked to lower guilt-proneness. From a socio-cognitive perspective, this pattern may be explained by differences in self-reflective processes. Difficulties in accurately predicting the feelings and behaviors of others may lead to a heightened focus on the self, which is characteristic of shame (Yang et al., 2010). Conversely, proneness to guilt may be related to advanced ToM skills (Davidson et al., 2017), as a deeper understanding of others’ perspectives can motivate the reparative and prosocial actions associated with guilt. Alternatively, peripheral physiological regulatory processes may offer a complementary explanation (Chiu et al., 2024; Ip et al., 2024). For instance, Ip et al. (2024) recently demonstrated that resting heart rate variability (HRV), a biomarker of emotion regulation, showed positive correlations to guilt-proneness, but not shame-proneness. Given the established association between higher autistic traits and lower emotion regulation abilities (e.g., Cai et al., 2019), it is plausible that reduced adaptive regulation in these individuals hinders the experience of guilt. At the same time, this diminished regulatory capacity may increase the likelihood of the negative self-evaluations central to shame. It is evident that multiple pathways likely link autistic traits and self-conscious emotions, a complex relationship that warrants further research.

Our study extends past findings by demonstrating that the differential association of shame/guilt and autistic trait is replicable across two other common autistic trait measures.

More importantly, replication of these associations provided a foundation for us to test our hypothesis of whether functional connectivity of the CMS could be a neurobiological mechanism explaining these profiles of SCE. Supporting our hypothesis, the mediation analysis revealed that autistic traits had indirect effects on shame-proneness through rFP functional connectivity with the PC, PCC and ACC. For guilt-proneness, autistic traits exerted its effects via rFP functional connectivity with the PC, but not the ACC and PCC. The rFP-rSFG functional connectivity was also a significant mediator of autistic traits with both shame- and guilt-proneness. Thus, our findings suggest that functional connectivity differences in brain regions linked to social cognition, that is, the CMS, in turn, underpins the differential association with shame- and guilt-proneness.

The ACC has been implicated in internal monitoring of self-referential stimuli (Northoff & Bermpohl, 2004) and social pain (Masten et al., 2011), while the PCC is thought to be important for the self-representation (Leech et al., 2011; Davey et al., 2016), and the integration of self-referential stimuli to the self (Northoff & Bermpohl, 2004). The FP-PCC functional network has recently been shown to be implicated in digesting complex information (Law et al., 2023). The brain regions identified in our mediation analyses, that is, PC, PCC, ACC, and SFG, have all shown atypical connectivity patterns in autistic individuals (Assaf et al., 2010; Yerys et al., 2015; Cheng et al., 2015). While we had hypothesized the role of the CMS as an underlying mechanism due to the difficulties in ToM/perspective taking in autistic individuals, we recognize that the functional significance of these regions needs to be carefully interpreted, given that we did not have specific measurements of ToM or social cognition. To date, only a handful of studies have examined whether ToM abilities in autistic individuals could be a mechanism for the differential experience of shame and guilt, but the results are mixed. While group comparisons did not reveal differences in ToM abilities between adults with high and low autistic traits, Davidson et al. (2017) reported that individual differences in performance on the Faux Pas test (Stone et al., 1998), predicted shame-proneness only in the group with high autistic traits. Similarly, Davidson et al. (2018) showed that performance on the Strange Stories Test (Happe, 1994), was positively correlated with proneness to guilt in autistic children but not in NT children. However, other studies have not replicated such associations. Li et al. (2023) found no associations between understanding of false beliefs and other people’s emotions with expressions of shame and guilt in autistic and NT children. van Trigt et al. (2023) directly tested ToM as a mediator of autistic traits and SCEs. They found that children with higher autistic traits showed lower ToM, but this did not in turn, associate with shame- or guilt-proneness.

One possible interpretation is that CMS connectivity disruptions in these regions contribute to difficulty in reflecting about how a “wrong” action has affected others in relation to their own behavior, which contributes to lower guilt-proneness, and less motivation to engage in reparative actions for their behavior for individuals with high autistic traits. However, connectivity disruptions may also contribute to dysregulations in over-evaluating the self, which increases shame-proneness. Alternatively, these patterns of connectivity could reflect the involvement of empathic processes, as recent meta-analyses have shown considerable overlap between ToM and empathy networks, which include similar regions like the ACC and PCC (Schurz et al., 2020; Maliske et al., 2023). In the past, ToM and empathy may have been considered as independent “cognitive” versus “affective” routes to understanding others (Kanske, 2018), but one can imagine that in processing real-life social interactions, particularly in contexts that elicit negative SCEs, both types of processing are likely required. Given that this is the first study to examine the neurobiological underpinnings of autistic traits and negative SCEs, our identification of these functional connectivity differences was exploratory in nature and future research is required to tease apart the aspects of cognitive and affective self-referential processing. Indeed, other mechanisms such as poor interoception (DuBois et al., 2016) and alexithymia (i.e., difficulties in identifying one’s emotions) (Bird & Cook, 2013) could be alternative pathways to the differences in shame- and guilt-proneness; especially because these aspects are implicated in reduced self-awareness among autistic individuals.

Several limitations hamper the findings of this study. First, our study design was essentially non-experimental and cross-sectional; although mediation analysis was used, causal relationships between the variables cannot be warranted. Second, we did not have an explicit measure of ToM/perspective taking or social cognition, which would help relate the functional connectivity differences with behavioral data. Third, our sample size did not allow us to examine gender-related differences to the experience of shame and guilt. Fourth, all the participants in the study were neurotypical. Future studies are encouraged to replicate these findings by including individuals diagnosed with autism, so that the full spectrum of autistic traits can be accounted for when examining its association with negative SCEs. Despite these limitations, this study contributes to a better understanding of the biological mechanism that underlie SCE processing. As shame and guilt are known to play roles in mood disorders such as depression (Pulcu et al., 2014), understanding the neural correlates of negative SCEs is important for a better understanding of the development of mood pathologies in autism, and how these interact with core difficulties in social cognition and communication.

## Conclusion

A growing body of research increasingly focuses on understanding the brain bases of complex moral emotions, such as shame and guilt. Given the clinical implications of dysregulated SCE processing among individuals with autism or high autistic traits, there is an increasing need to understand the mechanisms that explain the link between autism and shame- and guilt-proneness. Taking a dimensional approach, this study investigated rs-FC among a sample of NT adults to test whether the CMS, thought to underlie social difficulties in autism, can mediate the effects of autistic traits on negative SCE experience. Our findings affirm the involvement of the mPFC and other CMS in self-referential processing, which are central to proneness to shame and guilt, and the social communication difficulties in autism.

## Supporting information

Ip et al. supplementary material Ip et al. supplementary material

## Supplementary material

To view supplementary material for this article, please visit https://doi.org/10.1017/pen.2025.10004

## Research transparency and reproducibility

The supporting data for the findings presented in this study are available upon request from the corresponding author.
