# Supplementary material for: Autistic traits and proneness to shame and guilt: The mediating role of functional connectivity of cortical midline structures
Source: Personal Neurosci. 2026 Apr 7;9:e2. doi: 10.1017/pen.2025.10004 (PMC13125281; doi:10.1017/pen.2025.10004)
Supplement: Ip et al. supplementary material [file S2513988625100047sup001.docx]

**Supplementary Material**

**
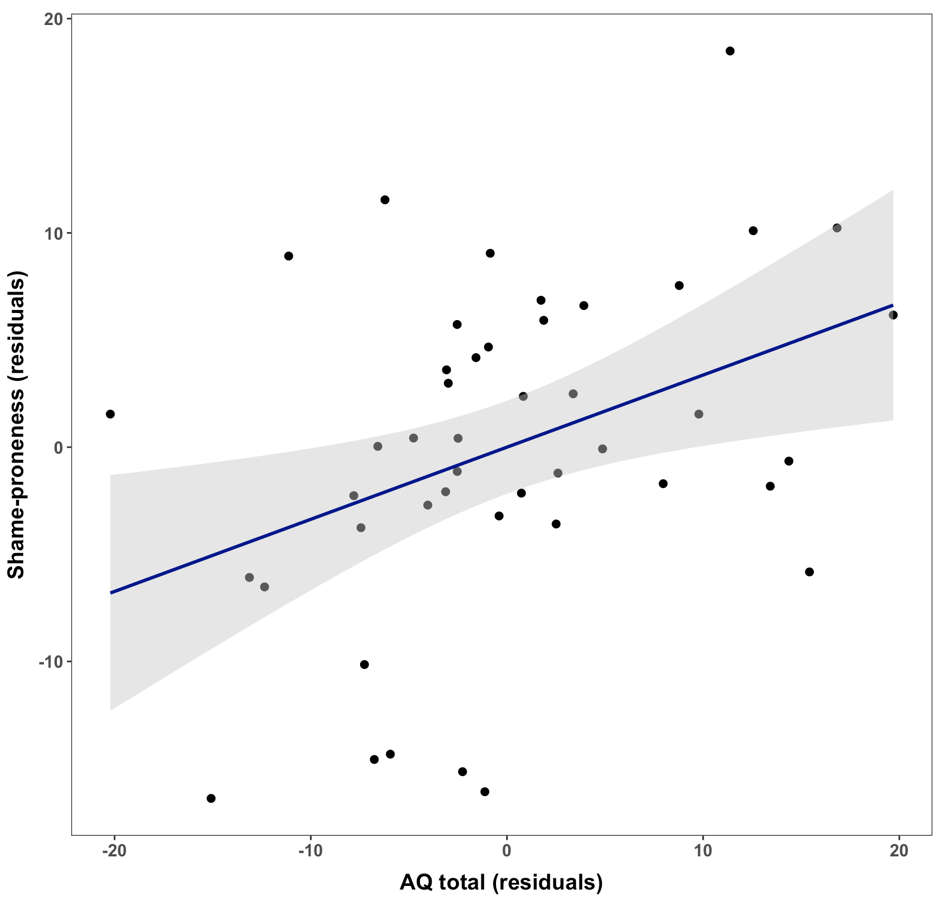
**

**S1 Fig.** Scatterplot showing the partial correlation between AQ and shame-proneness, controlling for guilt-proneness.

**
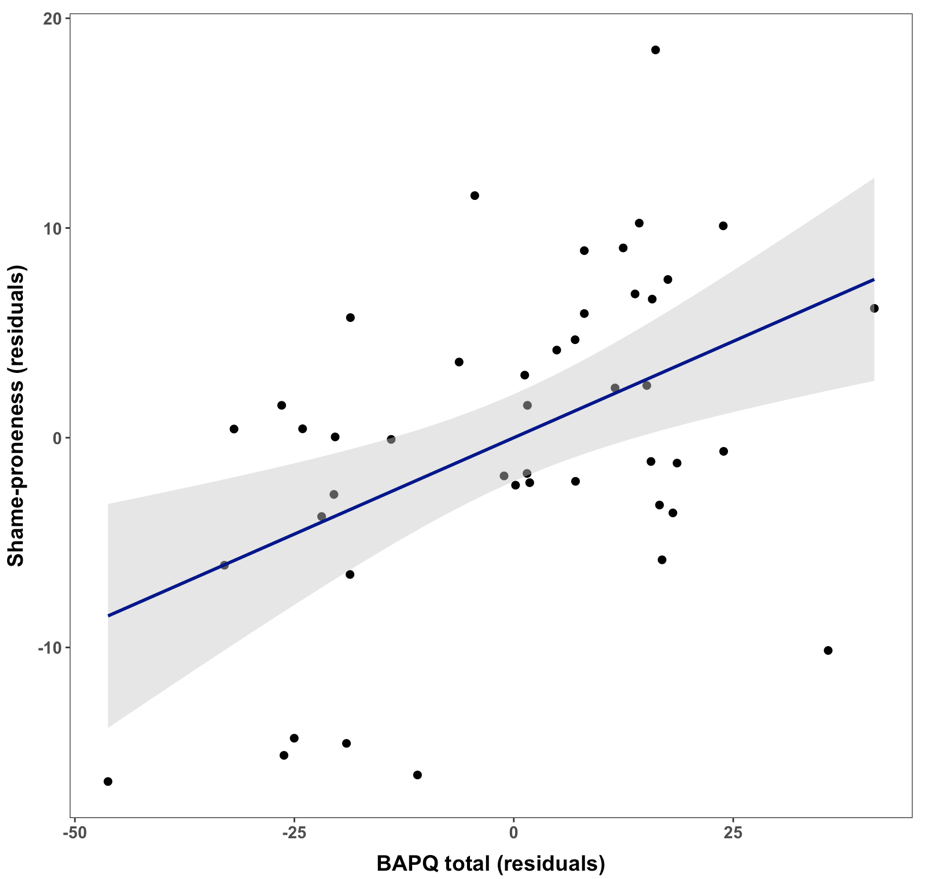
**

**S2 Fig.** Scatterplot showing the partial correlation between BAPQ and shame-proneness, controlling for guilt-proneness.

**
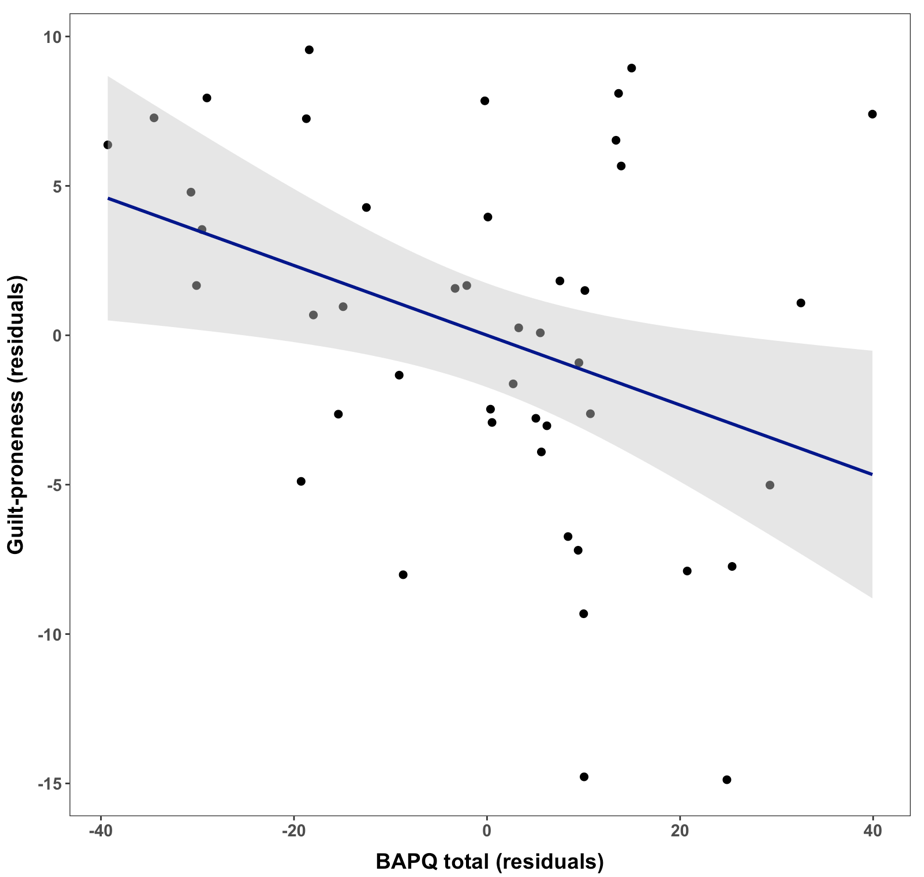
**

**S3 Fig.** Scatterplot showing the partial correlation between BAPQ and guilt-proneness, controlling for shame-proneness.
